# Physical exercise as treatment for adults with type 2 diabetes: a rapid review

**DOI:** 10.3389/fendo.2023.1233906

**Published:** 2023-09-28

**Authors:** Andressa Karoline Pinto de Lima Ribeiro, Josilayne Patrícia Ramos Carvalho, Natáli Valim Oliver Bento-Torres

**Affiliations:** ^1^ Graduate Program in Attention and Clinical Study in Diabetes, Institute of Medical Sciences, Federal University of Pará, Belém, Pará, Brazil; ^2^ Neurodegeneration and Infection Research Laboratory, João de Barros Barreto University Hospital, Federal University of Pará, Belém, Pará, Brazil; ^3^ Graduate Program in Human Movement Sciences, Institute of Health Sciences, Federal University of Pará, Belém, Brazil

**Keywords:** diabetes mellitus, physical exercise, rehabilitation, glycemic control, primary health care, quality of life, physical fitness, Noncommunicable diseases

## Abstract

**Background:**

Type 2 diabetes mellitus (T2DM) is a leading cause of disability-adjusted life years (DALY). Physical exercise is an effective non-pharmacological intervention to promote glycaemic control in T2DM. However, the optimal exercise parameters for glycemic control in individuals with T2DM remain unclear.

**Objective:**

This study aimed to analyze the relationship between physical training variables – frequency, intensity, type, duration, volume, and progression – and glycemic control in individuals with T2DM.

**Methods:**

A rapid systematic literature review was conducted on PubMed and LILACS databases. The PICOT strategy was employed to define the inclusion criteria. Eligible studies had to assess the impact of exercise parameters (frequency, intensity, type, duration, volume, and progression) on glycemic control indicators, primarily glycosylated hemoglobin (HbA1c). Randomized and non-randomized clinical trials were included in the review. The methodological quality of each study was assessed using the PEDro scale (PROSPERO - CRD 42021262614).

**Results:**

Out of 1188 papers initially identified, 18 reports met the inclusion criteria and were included in the analysis. A total of 1,228 participants with T2DM (1086 in exercise groups) were included in the selected studies. Among these studies, 16 (88.9%) were RCTs and 2 (11.1%) were nRCTs. The age of participants ranged from 43.1 and 68.9 years, and the average intervention duration was 16.8 weeks. Data on adherence to the intervention, adverse events, detailed intervention protocol, and its impacts on glycaemic control, lipid profile, blood pressure, anthropometric measures, medication, body composition, and physical fitness are reported.

**Conclusion:**

The evidence supports the safety and effectiveness of physical exercises as non-pharmacological interventions for glycemic control. Aerobic, resistance and combined training interventions were associated with reductions in HbA1c and fasting glucose. The diversity of the physical exercise intervention protocols investigated in the studies included in this review is an important limitation to generalizing evidence-based practice. The call for action is mandatory to implement large-scale education programs on the prevention of diabetes and public health policies aimed to include well-planned and supervised exercise programs as an essential part of the primary prevention of type 2 diabetes.

**Systematic Review Registration:**

PROSPERO, identifier (CRD42021262614).

## 1 Introduction

The prevalence of diabetes worldwide is rapidly increasing, with estimates suggesting a rise from 536.6 million adults (20–79 years) in 2021 to 783.2 million in 2045 (1). Type 2 diabetes mellitus (T2DM) is the third leading cause of increased disability-adjusted life years (DALY) among individuals aged 50 to 74 (2). Diabetes is associated with various complications such as blindness, kidney failure, heart attacks, stroke, and lower limb amputation. Furthermore, the age-standardized DALYs caused by T2DM in the Americas in 2019 were 29% greater than the global burden and increased by 27.4% from 1990 onwards (3). Diabetes is an economic burden, challenging public health policies worldwide. The direct costs associated with hospitalizations, outpatient procedures, and diabetes medications reached USD 966 billion in 2021 (1). Type 2 diabetes is responsible for an average of 5.4 quality-adjusted life-years (QALYs) lost and for limitations on occupational and daily activities (2, 4).

The treatment of T2DM requires physical exercise, a balanced diet, and medication (5). Well-planned, evidence-based, and supervised physical exercise is a cost-effective therapeutic strategy for managing T2DM, reducing insulin resistance, improving muscle glucose utilization, enhancing insulin sensitivity (6), and increasing QALY (7). Furthermore, exercise offers additional health benefits, including decreased cardiovascular disease risk, enhanced physical fitness, weight maintenance, and improved mental well-being and quality of life for individuals with diabetes (8–10).

Physical exercise should be tailored to meet the specific personal and clinical needs of each patient (11). An individualized exercise program is crucial for therapeutic success, although there are general guidelines (9, 12–16). Properly prescribed and executed physical exercise offers significant benefits to individuals with T2DM, serving as an effective tool for metabolic management and a non-pharmacological strategy for the prevention and treatment of T2DM in adults and older adults (9, 13). The dose of physical training, encompassing frequency, intensity, type, duration, volume, and progression of exercise, plays a pivotal role in determining the extent of the training response (5, 17–21).

Frequently, there is a lack of detailed information in papers regarding exercise training parameters to estimate the exercise dose-response, along with methodological differences between studies (22). This scenario limits our understanding of the role that frequency, intensity, type, duration, volume, and progression of the exercise play an effective glycemic control. Also, the lack of evidence-based information for the exercise professional compromises the individualized training protocols for individuals with T2DM. The primary objective of this study was to analyze the relationship between the variables of aerobic, resistance, and combined physical training and glycemic control in individuals with T2DM through a rapid systematic literature review.

## 2 Methods

We conducted a rapid literature review to investigate the effects of physical exercise interventions on glycemic control in adults with Type 2 Diabetes Mellitus (T2DM). We analyzed the exercise parameters of frequency, intensity, type, duration, volume, and progression. The review protocol was registered at PROSPERO (International Prospective Register of Systematic Reviews) under number CRD 42021262614. We followed the PRISMA Guideline (23) to report the results.

### 2.1 Eligibility criteria

We used the PICOT strategy to define the eligibility criteria. *Population*: Studies with Type 2 Diabetes Mellitus participants aged 45 years or older. *Intervention*: Physical exercise interventions, including aerobic, resistance, or combined exercises, with at least one modifiable variable in the individualized exercise prescription (frequency, intensity, type, duration, volume, or progression). *Comparison*: T2DM participants on different types of physical training or usual diabetes care. *Outcome:* The impact of modifiable exercise variables on glycosylated hemoglobin (HbA1c) and additionally on other glycemic control indicators. *Type of Study*: Randomized and non-randomized clinical trials. Only articles published in English or Portuguese, between 2012 and February 2023, were included.

Exclusion criteria: We excluded systematic reviews, meta-analyses, and observational studies. Studies that did not assess HbA1c, interventions exclusively based on education for an active lifestyle or mind-body therapies (except Pilates), unpublished studies, and gray literature were also excluded.

### 2.2 Information sources and search strategy

We conducted searches in the PubMed and LILACS databases. We used the following search strategy: (“Diabetes Mellitus Type 2” OR Diabetes OR DM2 OR “Diabetes Mellitus, Type 2”) AND (“Physical Exercise” OR “Circuit-based Exercise” OR “Resistance Training” OR “Aerobic Training” OR Exercise OR “Resistance Training” OR “Circuit-based Exercise”). The searches were conducted until February 2023.

### 2.3 Data selection

The study selection process consisted of four stages: Identification, screening by title and abstract, eligibility assessment, and inclusion. During the identification stage, we collected all papers found during the search process and searched for duplicates. At the screening stage, we analyzed the titles, abstracts, and keywords of the identified studies and excluded articles that did not meet the selection criteria. Two authors independently reviewed each record retrieved from the search, and articles that clearly did not meet the criteria were eliminated.

In the eligibility assessment stage, we conducted a full read of the articles to confirm their suitability for inclusion. Each article was independently read by two researchers, and any divergences regarding eligibility were discussed in a consensus meeting. Divergence occurred in only seven (8.86%) of the articles read in full, and two papers were included in the review after discussion (24, 25). We used the Zotero software for reference management.

### 2.4 Data collection process

Two authors independently extracted the following data, when available: authorship, publication date, study design details, sample size, age, sex proportion, inclusion criteria for participants, exercise variables (frequency, intensity, type, duration, volume, and progression), monitoring strategy, outcomes, follow-up time, and losses. If described, additional data such as diet, supervision, schedule, adherence, medication, and adverse effects were also collected.

Glycemic control through HbA1c measurement was considered the primary outcome of interest, with additional variables of interest including fasting and postprandial blood glucose, serum lipids and fractions, blood pressure, anthropometry/body composition, physical fitness, and medication changes.

### 2.5 Study risk of bias assessment

The methodological quality of each study was analyzed using the PEDro scale (Physiotherapy Evidence Database) (26). The assessment was based on the information described in each study. In case of doubt or missing information, the criterion was qualified as not meeting the PEDro scale recommendation. The final score on the PEDro scale is the sum of the number of criteria classified as satisfactory among criteria 2 to 11. Criterion 1, which assesses the study’s external validity, is not considered in the final score. Two independent researchers analyzed each clinical trial, and there was no disagreement on any evaluation item. For clinical trials indexed on PEDro, the database score was used (12, 24, 25, 27–35). PEDro score was not used as an exclusion criterion.

## 3 Results

### 3.1 Study selection

**Figure 1:**
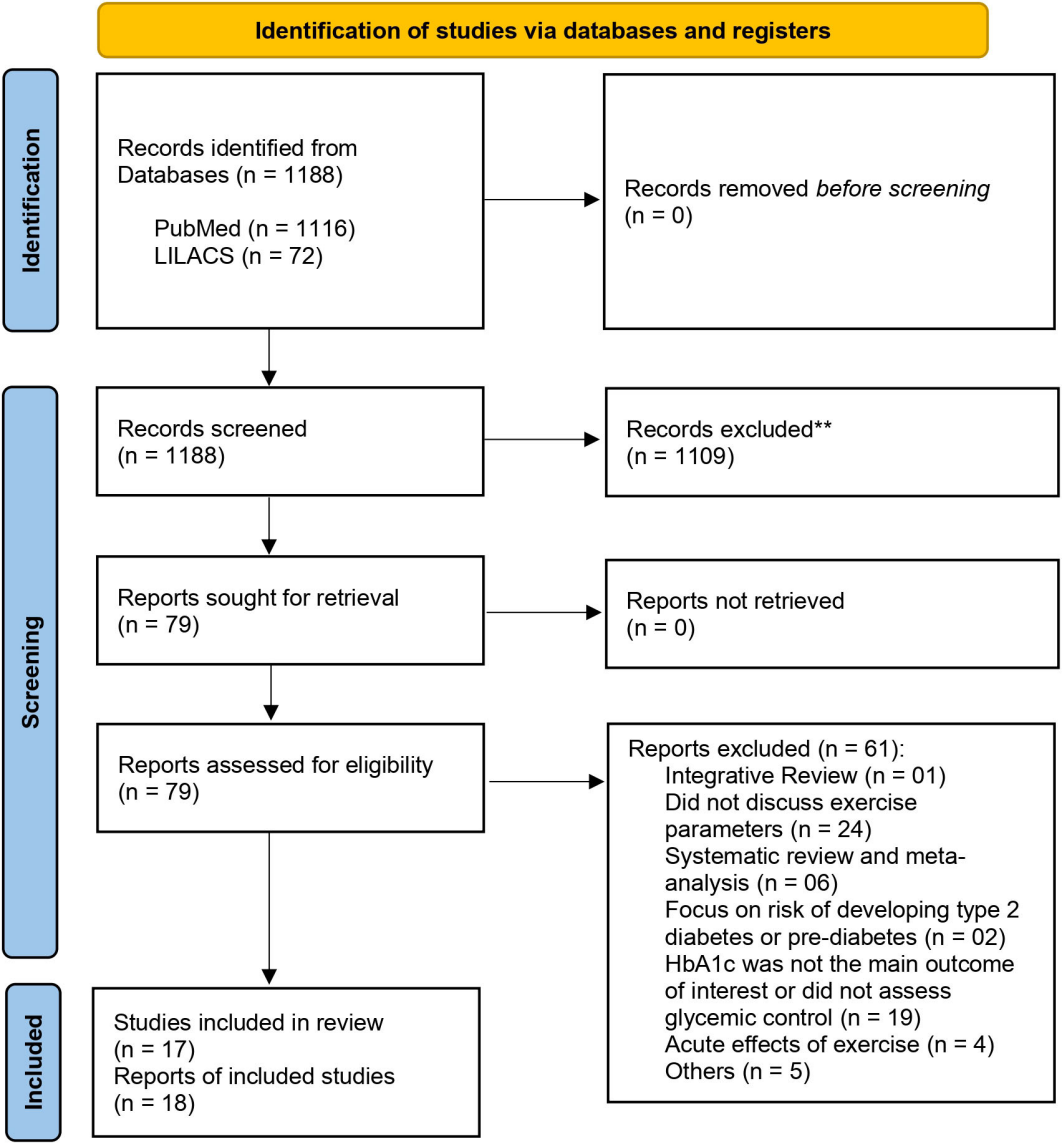
Flow diagram of studies selection and included in this review.

Initially, a total of 1188 records were identified, with no duplicates. After reviewing titles and abstracts, 79 articles were selected for full reading. Among these, 61 were excluded, and 18 articles met our inclusion criteria (12, 24, 25, 27–41). The detailed selection process and reasons for exclusion are described in **Figure 1**.

### 3.2 Characteristics of the included studies

A total of 1,228 participants with T2DM, aged between 43.1 and 68.9 years, were included in the studies. Among them, 1086 were allocated in exercise groups and 412 were allocated in non-exercised control groups. Sixteen of the included studies (88.9%) were randomized clinical trials, and 2 (11.1%) were non-randomized clinical trials, published between 2012 and 2022. The intervention length ranged from 10 to 52 weeks, with 66.67% of the interventions lasting between 12 and 16 weeks. Eight studies (44.4%) reported no adverse events during the intervention, four (22.2%) reported some minor events (such as back pain, tendinitis, hypoglycemia, or muscle injury), and six (33.3%) did not provide information on adverse events. The characteristics of the included studies are presented in **Table 1**.

**Table 1:** Characteristics of clinical trials included in this review.

Author, year	Type of study	Participants Characteristics (Baseline)	Intervention time/Adherence to the intervention	Adverse events
Alvarez et al., 2016 (27)	RCT	**Low volume, high-intensity interval training group:** 45.6 ± 3.1 years of age; 30.6 ± 1 kg/m².	16 weeks/89 ± 5%	No adverse events were reported.
		**Non-exercise control group:** 43.1 ± 1.5 years of age. 30.4 ± 0.4 kg/m².		
Andrade et al., 2016 (37)	nRCT	**Moderate-intensity aerobic training group**: 51.72 years of age; HbA1c: 5.6 ± 1.8%	12 weeks/No information available	Hypoglycemia
Bacchi et al., 2012 (28)	RCT	**Moderate-intensity aerobic training group:** 57.2± 1.6 years of age; 29.5 ± 1.1 kg/m²; HbA1c: 7.29± 0.15%.	16 weeks/Aerobic training: 93%; Resistance Exercise: 89%	Back pain (n=4); tendinitis (n=1); asymptomatic hypoglycemia (n=17)
		**Resistance exercise group:** 55.6 ± 1.7 years of age; 29.2 ± 1.0 kg/m²; HbA1c: 7.30± 0.16%.		
Banitalebi et al., 2019 (29)	RCT	**HIIT group:** 55.36 ± 5.94 years of age; 29.27 ± 3.0 kg/m²; HbA1c: 9.6± 1.1%.	10 weeks/HIIT: 78%; HIIT + Resistance exercise: 82%.	No adverse events were reported.
		**Combined group:** 54.14 ± 5.43 years of age; 28.68 ± 4.34 kg/m²; HbA1c: 9.5± 0.9%.		
		**Non-exercise control group:** 55.71± 6.40 years of age; 30.12 ± 3.52 kg/m²; HbA1c 9.0 ± 0.5%.		
Banitalebi et al., 2021 (25)	RCT	**HIIT group:** 55.36 ± 5.94 years of age; 29.29 ± 3.19 kg/m²; HbA1c: 9.64± 1.01%.	10 weeks/HIIT: 78%; HIIT + resistance exercise: 82%.	No adverse events were reported.
		**Combined group:** 54.14 ± 5.43 years of age; 30.57 ± 2.97 kg/m²; HbA1c: 9.49± 0.85%.		
		**Non-exercise control group:** 55.71± 6.40 years of age; 30.12 ± 3.52 kg/m²; HbA1c: 9.0 ± 0.5%.		
Cassidy et al., 2019 (24)	RCT	**HIIT group:** 60 ± 3 years of age. 31.2 ± 1.70 kg/m². HbA1c: 7.13 ± 0.31%.	12 weeks/Full participation	No information available
		**Non-exercise control group**: 59 ± 3 years of age. 32.0 ± 1.65 kg/m². HbA1c: 7.18 ± 0.17%.		
Chien et al, 2022 (36)	RCT	**Resistance exercise group:** 67.6 ± 7.7 years of age. 24.3 ± 3.4 kg/m². HbA1c: 8.1 ± 1.1%.	12 weeks/Resistance exercise: 82%.	No adverse events were reported.
		**Non-exercise control group:** 67.3 ± 6.1 years of age. 25.5 ± 3.7 kg/m². HbA1c 7.6 ± 0.6%.		
Gholami et al., 2020 (30)	RCT	**Moderate-intensity aerobic training group:** 53.4 ± 9.1 years of age; 28.2 ± 2.5 kg/m².	12 weeks/> 90%	No adverse events were reported.
		**Non-exercise control group:** 52.2 ± 8.5 years of age; 28.7 ± 1.8 kg/m².		
Kong et al, 2022 (38)	RCT	**Moderate-intensity aerobic training group:** 50 ± 10 years of age; 28 ± 5 kg/m²; HbA1c: 9.04 ± 2.13%.	16 weeks/No information available	No information available
		**Non-exercise control group:** 50 ± 8 years of age. 29 ± 4 kg/m²; HbA1c: 8.86 ± 1.81%.		
Li et al., 2012 (39)	RCT	**High intensity aerobic exercise:** 50.3 ± 1.2 years of age; 26.1 ± 0.7 kg/m²; HbA1c: 6.6 ± 0.2%.	12 weeks/No information available	No information available
		**Low intensity aerobic training:** 52.0 ± 1.3 years of age; 25.9 ± 0.6 kg/m²; HbA1c: 6.7 ± 0.2%.		
Magalhães et al., 2019 (31)	RCT	**Combined - HIIT + Resistance training group:** 56.7 ± 8.3 years of age; 30.1 ± 5.7 kg/m²; HbA1c: 6.9 ± 1.1%.	52 weeks/HIIT + Resistance: 86.8%; Moderate intensity + resistance: 86.2%	Muscle injuries (n=3)
		**Combined - Moderate-intensity aerobic + Resistance training group:** 59.7 ± 6.5 years of age; 31.1 ± 5.0 kg/m²; HbA1c: 7.4 ± 1.9%.		
		**Non-exercise control group:** 59.0 ± 8.1 years of age; 30.7 ± 5.0 kg/m²; HbA1c: 7.4 ± 1.8%.		
Mitranun et al., 2014 (32)	RCT	**HIIT group**: 61.7 ± 2.7 years of age; 29.4 ± 0.7 kg/m²; HbA1c: 7,73 ± 2.3%.	12 weeks/No information available	No information available
		**Moderate-intensity aerobic training group:** 61.2 ± 2.8 years of age; 29.6 ± 0.5 kg/m²; HbA1c: 7,64 ± 2.3%.		
		**Non-exercise control group:** 60.9 ± 2.4 years of age; 29.7 ± 0.4 kg/m²; HbA1c: 7.82 ± 2%.		
Nicolucci et al., 2012 (12)	RCT	**Combined group:** < 60 years of age. n=163. e ≥ 60 years of age. n=140; 31.2 ± 4.6 kg/m²; HbA1c: 7.12 ± 1.4%.	52 weeks/No information available	Adverse events reported
		**Non-exercise control group:** < 60 years of age. n=163. e ≥ 60 years of age. n=140; 31.9 ± 4.6 kg/m²; HbA1c: 7.15 ± 1.4%.		
Pandey et al., 2017 (33)	RCT	**Continuous high-intensity exercise group (BURST):** 68 ± 9 years of age; 32.3± 2.1 kg/m²; HbA1c: 8.14 ± 0.49%.	12 weeks/increased adherence on BURST group	No adverse events were reported.
		**Moderate-intensity aerobic training group:** 65 ± 9 years of age; 32.4± 1.9 kg/m²; HbA1c: 8.18 ± 0.35%.		
Sabag et al., 2020 (34)	RCT	**HIIT group:** 56.9 ± 2.1 years of age; 37.5 ± 1.6 kg/m²; HbA1c: 7.1 ± 0.4%.	12 weeks/Aerobic: 93%; HIIT: 98%; Control: 63%	No adverse events were reported.
		**Moderate-intensity aerobic training group:** 54.8 ± 2.4 years of age; 34.3 ± 1.1 kg/m²; HbA1c: 7.3 ± 0.4%.		
		Exercise control group*: 51.9 ± 1.4 years of age; 35.8 ± 1.7 kg/m²; HbA1c: 7.6 ± 0.5%.		
Stoa et al., 2017 (40)	nRCT	**HIIT group:** 59 ± 11 years of age; 32.0 ± 4.7 kg/m²; HbA1c: 7.78 ± 1.39%.	12 weeks/No information available	No information available
		**Moderate-intensity aerobic training group:** 59 ± 10 years of age; 31.1 ± 4.5 kg/m²; HbA1c: 6.84 ± 0.88%.		
Winding et al., 2018 (41)	RCT	**HIIT group:** 54 ± 6 years of age; 28.1 ± 3.5 kg/m²; HbA1c: 6.8 ± 0.8%.	11 weeks/HIIT: 91%; Aerobic: 94%.	No adverse events were reported.
		**Moderate-intensity aerobic training group:** 58 ± 8 years of age; 27.4 ± 3.1 kg/m²; HbA1c: 6.9 ± 0.9%.		
		**Non-exercise control group:** 57 ± 7 years of age; 28.0 ± 3.5 kg/m²; HbA1c: 7.0 ± 1.15%.		
Yang et al., 2017 (35)	RCT	**Combined -** Low intensity, low volume resistance training (walking or cycling): 52.2 ± 1.0 years of age. 32.5 ± 0.9 kg/m²; HbA1c: 7.6 ± 0.3%	20 weeks/No information available	No information available
		**Combined -** High intensity, low repetition resistance training + aerobic (walking or cycling): 49.8 ± 1.4 years of age; 30.2 ± 0.7 kg/m²; HbA1c: 7.7 ± 0.2%		
		**Combined** - Low intensity, high repetition resistance training + aerobic (walking or cycling): 54.6 ± 1.2 years of age; 32.4 ± 0.8 kg/m²; HbA1c: 7.4 ± 0.3%.		

RCT, Randomized Clinical Trial; nRCT, Non-randomized clinical trial; HIIT, High-intensity interval training. BURST, continuous high-intensity exercise; * participants in the control group performed supervised sessions every 2 weeks involving a 5-min low-intensity cycle ergometer and were prescribed a fitball exercise and upper and lower body stretches. General characteristics of the participants at baseline, intervention time, adherence to the intervention and adverse events are described.

### 3.3 Risk of bias in studies

Four articles (22.2%) were considered to have a high risk of bias with scores below 5 on the PEDro scale (12, 24, 37, 40). Fourteen articles (77.8%) had moderate to high methodological quality, with scores ≥ 5 on the PEDro scale (25, 27–36, 38, 39, 41) (**Table 2**).

**Table 2:** Risk of bias assessment - PEDro Scale.

PEDro criteria	1	2	3	4	5	6	7	8	9	10	11	Total Score
Author, year												
Alvarez et al., 2016 (27)	Y	Y	N	Y	N	N	Y	Y	N	Y	Y	6/10
Andrade et al., 2016 (37)	Y	N	N	N	N	N	N	N	N	N	Y	1/10
Bacchi et al., 2012 (28)	Y	Y	Y	Y	N	N	Y	Y	N	Y	Y	7/10
Banitalebi et al., 2019 (29)	Y	Y	Y	Y	N	N	N	N	N	Y	Y	5/10
Banitalebi et al., 2021 (25)	Y	Y	Y	Y	N	N	N	N	N	Y	Y	5/10
Cassidy, et al., 2019 (24)	Y	Y	Y	Y	N	N	N	N	N	Y	N	4/10
Chien et al., 2022 (36)	Y	Y	N	Y	N	N	Y	Y	Y	Y	Y	7/10
Gholami et al., 2020 (30)	N	Y	N	Y	N	N	Y	Y	N	Y	Y	5/10
Kong et al., 2022 (38)	Y	Y	N	Y	N	N	Y	Y	Y	Y	Y	7/10
Li et al., 2012 (39)	Y	Y	Y	Y	N	N	N	Y	Y	Y	Y	7/10
Magalhães et al., 2019 (31)	Y	Y	N	Y	N	N	N	N	Y	Y	Y	5/10
Mitranun et al., 2014 (32)	Y	Y	N	Y	N	N	N	Y	N	Y	Y	5/10
Nicolucci et al., 2012 (12)	Y	Y	N	N	N	N	N	Y	N	Y	Y	4/10
Pandey et al., 2017 (33)	N	Y	N	Y	N	N	N	Y	N	Y	Y	5/10
Sabag et al., 2020 (34)	N	Y	Y	Y	N	N	N	Y	Y	Y	Y	7/10
Stoa et al., 2017 (40)	Y	N	N	N	N	N	N	Y	Y	Y	Y	4/10
Winding et al., 2018 (41)	N	Y	Y	Y	N	N	N	Y	Y	Y	Y	7/10
Yang et al., 2017 (35)	Y	Y	N	Y	N	N	Y	N	Y	Y	Y	6/10

Y, Yes; N, No. Criteria: 1 - the eligibility criteria were specified (This item is not used to calculate the PEDro score); 2 - subjects were randomly allocated to groups; 3 - allocation was concealed; 4 - the groups were similar at baseline regarding the most important prognostic indicators; 5 - there was blinding of all subjects; 6 - there was blinding of all therapists who administered the therapy; 7 - there was blinding of all assessors who measured at least one key outcome; 8 - measures of at least one key outcome were obtained from more than 85% of the subjects initially allocated to groups; 9 - all subjects for whom outcome measures were available received the treatment or control condition as allocated or, where this was not the case, data for at least one key outcome was analyzed by “intention to treat”; 10 - the results of between-group statistical comparisons are reported for at least one key outcome; 11 - the study provides both point measures and measures of variability for at least one key outcome.

### 3.4 Characteristics of intervention protocols

Most articles reported the use of aerobic exercise as the primary intervention method. Ten studies utilized high-intensity interval training (HIIT) exercises, while ten used moderate-intensity exercises, primarily through ergometers such as treadmills and cycle ergometers. The duration of sessions varied from 15 to 90 minutes. Twelve papers (66.67%) reported to monitor exercise intensity: Nine used a heart rate monitor, two used a perceived exertion scale, and one used palpation of the carotid artery. In terms of frequency, eleven (78.6%) studies performed aerobic exercises three times a week, two (14.3%) studies five times a week, while one study reported performing three to five times a week without describing specific criteria (see **Table 3**).

**Table 3:** Physical exercise protocols. Information on modality, type of exercise, duration, intensity, monitoring of intensity, frequency, and volume.

Author, year	Modality/Exercise type	Intervention Protocol			
		Number of sets, repetitions and intervals (Resistance)/Duration of the session (Aerobic)/Progression	Intensity/monitoring	Frequency	
Alvarez et al., 2016 (27)	Progressive HIIT, with intervals of high-intensity exercise (running/jogging) interspersed with low-intensity active recovery (walking): n=13	Week 0-4: 8 x 30-34 sec.	Running: 90-100% HHR. Walking: <70% HHR; Objective monitoring (heart rate monitor)	3x/week.	
		Rest: 9 x 120 sec.			
		Week 5-9: 10 x 38 a 44 sec.			
		Rest: 11 x 108 sec.			
		Week 10-13: 12 x 46 a 50 sec.			
		Rest: 13 x 100 sec.			
		Week 14-16: 14 x 52 a 58 sec.			
		Rest: 15 x 96 sec.			
	Non-exercise control group: n=10				
Andrade et al., 2016 (37)	Moderate-intensity aerobic training group (walking): n=25	50 minutes	50-60% HHR; Objective monitoring (heart rate monitor)	3x/week.	
Bacchi et al., 2012 (28)	Moderate-intensity aerobic training group (cardio training equipment): n=19	60 minutes	60-65% HHR	3x/week.	
			Objective monitoring (heart rate monitor)		
	Resistance training (free weights and weight machines): n=19	60 minutes	Adaptation phase: 30-50% 1RM. Gradual increase to 70-80% 1RM	3x/week.	
		9 exercises -3 sets of 10 reps each exercise.			
Banitalebi et al., 2019 (29)	Combined training (aerobic: treadmill or cycle ergometer + resistance: strength training machines): n=14	Aerobic + resistance: 50 min	Week 1-2: 60% HR max.	3x/week.	
		Week 1-2: 20 min.	Week. 3-10: 70% HR max.		
		Week 3-10: 30 min.	Objective monitoring (heart rate monitor)		
		Resistance training: 8 exercises			
		Week 1-2: 1x15 RM;			
		Week 3-10: 2 to 3 sets of 10-15 RM			
	High-intensity aerobic interval training (HIIT) (cycle ergometer): n=14	5 min warm-up; 4×30 sec cycles. interspersed with 2 min of recovery; 4 minutes of cool down.	Maximum intensity and power adjusted based on participants’ performance and perceived effort.	3x/week.	
	Non-exercise control group: n=14				
Banitalebi et al., 2021 (25)	Combined training (aerobic: treadmill or cycle ergometer + resistance: strength training machines): n=14	Aerobic + resistance: 50 min	Week 1-2: 60% HRmax.	3x/week.	
		Week 1-2: 20 min	Week. 3-10: 70% HR max.		
		Week 3-10: 30 min	Objective monitoring (heart rate monitor)		
		Resistance training: 8 exercises			
		Week 1-2: 1x15 RM.			
		Week 3-10: 2 to 3 sets of 10-15 RM.			
	High-intensity aerobic interval training (HIIT) (cycle ergometer): n=14	5 min warm-up, 4×30 sec cycles interspersed with 2 min of recovery; 4 minutes of cooldown.	Maximum intensity and power adjusted based on participants’ performance and perceived effort.	3x/week.	
	Non-exercise control group: n=14				
Cassidy et al., 2019 (24)	High-intensity aerobic interval training (HIIT) (exercise bike): n=11.	5 min warm-up with perceived exertion (RPE) from 9 (‘very light’) to a (a little difficult’). Five sets at 16-17 RPE (‘pretty hard’). Each set was interspersed with a 3 min recovery period. 3 min cooldown.	Perceived exertion scale.	3x/week.	
	Non-exercise control group: n=11	The duration of the intervals: 1st week - 2 min, increasing by 10 seconds each week.			
Chien et al., 2022 (36)	Resistance training: n=19.	5-10 min warm-up, 3 sets of 8 to 15 reps, 1 to 2 min rest. Total duration: 30 min.	Perceived exertion scale.	3x/week	
	Non-exercise control group: n=18.				
Gholami et al., 2020 (30)	Moderate-intensity aerobic training group (cycle ergometer): n=16	2 weeks of familiarization: 20 min.	2 weeks of familiarization: 50% HR reserve	3x/week.	
		12 weeks: 30-45 min.	12 weeks: 50-70% HR reserve		
	Non-exercise control group: n=15				
Kong et al., 2022 (38)	Moderate-intensity aerobic training group (calisthenic exercises): n= 35	60-90 min	60-70% HR Max.	3-5x/week	
	Non-exercise control group: n = 40				
Li et al., 2012 (39)	Low-intensity aerobic exercise (treadmill): n=27	Week 1: 1x15 min	Week. 1-2: 50% VO_2_peak	5x/week.	
		Week 2: 2x15 min	Week. 3-4: 50% VO_2_peak		
		Week. 3-4: 2x20 min	Week. 5-12: 50% VO_2_peak.		
		Week. 5-12: 2x120 kcal -on average 56,1 min	Objective monitoring (heart rate monitor)		
	High-intensity aerobic exercise (treadmill): n=28	Week 1: 1x15 min	Week. 1-2: 50% VO_2_peak	5x/week.	
		Week 2: 2x15 min	Week. 3-4: 65% VO_2_peak.		
		Week 3-4: 2x15 min.	Week. 5-12: 75% VO_2_peak.		
		Week. 5-12: 2x120 kcal - on average 34,3 min	Objective monitoring (heart rate monitor)		
Magalhães et al., 2019 (31)	Combined: High-Intensity Interval Training (HIIT) (cycling) + resistance training (RT), n=13	Calculated for both groups using the weekly target of energy expenditure (10 kcal/kg) and considering the individual VO2 max. Monthly update based on body weight, and every 3 months based on VO2 max, without modifications on the weekly target of 10 kcal/kg.	HIIT: Objective monitoring (heart rate monitor). Weeks 1-4: continuous exercise, 40-60% HHR	3x/week.	
			Weeks 5-6: 2-minute bouts at 70% HHR, followed by 1 minute at 40-60% HHR		
			Weeks 7-8: 2-minute bouts at 80% HHR, followed by 1 minute at 40-60% HHR		
		HIIT: 33.1 ± 6.4 min	Weeks 9-52: 1-minute bouts at 90% HHR, followed by 1 minute at 40-60% HHR		
		RT: 8 exercises - 1 set of 10-12 repetitions	Resistance training: The prescribed weight was increased once the participant was able to complete 12 repetitions for each set of exercises on two consecutive sessions.		
	Moderate-intensity continuous training (cycling) + resistance training (RT), n=16	Moderate-intensity training: 45.0 ± 7.1 min.	Aerobic 40-60% HHR - Objective monitoring (heart rate monitor)	3x/week.	
		RT: 8 exercises - 1 set of 10-12 repetitions	Resistance training: The prescribed weight was increased once the participant was able to complete 12 repetitions for each set of exercises on two consecutive sessions.		
	Non-exercise control group: n=22				
Mitranun et al., 2014 (32)	HIIT group (treadmill): n=14	Week 1-6: 30 min.	Week. 1-2: 50% VO_2_peak	3x/week.	
		Week 7-12: 40 min.	Week. 3-6: 60% VO_2_peak		
			Week. 7-12: 65% VO_2_peak		
	Moderate-intensity aerobic training group: (treadmill): n=14		Week. 1-2: 50% VO_2_peak	3x/week.	
			Week. 3-6:1 min. a 80% VO_2_peak e 4 min. a 50% VO_2_peak		
			Week. 7-12: 1 min on 85% VO_2_peak and 4 min on 60% VO_2_peak.		
	Non-exercise control group: n=15				
Nicolucci et al., 2012 (12)	Combined training (aerobic: treadmill, step, elliptical, arm ergometer or cycle ergometer/resistance: 4 exercises: bench press, lateral pulldown, leg press, and abdominal curl, or equivalent exercises targeting the same muscles): n= 278	75 min	The intensity was adjusted according to improvements in VO2 max and muscle fitness.	2x/week.	
	Non-exercise control group: n= 260				
Pandey et al., 2017 (33)	Moderate-intensity aerobic training group: (treadmills, exercise bikes, and outdoor walks): n= 19	30 min.	60% HRmax.	5x/week.	
			Heart rate monitoring by palpation of the carotid artery		
	Continuous high-intensity exercise group: (treadmills, exercise bikes, and outdoor walks): n= 21	3x10 min.	85% HRmáx	5x/week.	
			Frequency monitoring by palpation of the carotid artery		
Sabag et al., 2020 (34)	Moderate-intensity continuous exercise (cycling): n=12	40 to 55 min.	60% VO_2_peak	3x/week.	
	High-intensity interval training (HIIT): (cycling): n=12	Minimum 19 min.	90% VO_2_peak	3x/week.	
		10 min. warming up			
		4 min. HIIT			
		5 min. cooldown			
	Exercise control group: n=11				
Stoa et al., 2017 (40)	High-intensity interval training (HIIT) (outdoor walk or run): n=19	15 min warming up 70% Fcpeak	85-95% FCpeak	3x/week.	
		4x4 min at 90% HR peak with 3 min intervals at 70% HR peak	Objective monitoring (heart rate monitor)		
		12 min cooldown at 70%Fcpeak.			
	Moderate-intensity continuous exercise (MICT) (outdoor walk or run): n=19	60 min.	70-75% HRpeak	3x/week.	
			Objective monitoring (heart rate monitor)		
Winding et al., 2018 (41)	Moderate-intensity aerobic training group (cycling): n=12	5 min warm-up (at 40% of Wpeak) + 40 min.	50% of Wpeak.	3x/week.	
			Objective monitoring (heart rate monitor)		
	HIIT group: (ciclismo): n=13	5 min warm-up (at 40% of Wpeak) + 20 min.	1 min at 95% Wpeak and 1 min active recovery at 20% Wpeak.	3x/week.	
			Objective monitoring (heart rate monitor)		
	Non-Exercise control group: n=7				
Yang et al., 2017 (35)	Combined RT1 - Low intensity, low volume resistance training + aerobic (walking or cycling): n=16	10 exercises - 2 sets x 15RM	50% of 1 RM	5x/week	
		RT initiated after 3 months (50%)			
	Combined RT2 - High intensity, low repetition resistance training + aerobic (walking or cycling): n=17	10 exercises - 3 sets x 7RM	75% of 1 RM	5x/week	
		RT initiated at the beginning (100%)			
	Combined RT3 - Low intensity, high repetition resistance training + aerobic (walking or cycling): n=18	10 exercises - 2 sets x 15RM	50% of 1 RM	5x/week	
		RT initiated at the beginning (100%)			
			TA: 60-80% HHR or VO_2_peak		

HHR, Heart Rate Reserve; Hrmax, Maximum Heart Rate; sec., seconds; RT, Resistance training; HIIT, High-Intensity Interval Training; FC, Heart Rate; VO2, maximal oxygen consumption; FCLAn, Anaerobic heart rate limit.

Resistance training as an exclusive modality was performed in two studies (10.52%). One study employed 60-minute high-intensity resistance training, three times a week, utilizing free weights and weightlifting machines (28). In the second study, participants performed 30-minute, home-based unsupervised, moderate-intensity exercises, following instructions from a booklet, three times a week (36).

Five papers (27.8%) reported combined training, which involved both aerobic and resistance exercises as a treatment approach, including low, moderate, and high-intensity interventions. The aerobic component of the training was performed through activities such as cycling, walking, or using ergometers, while the resistance component utilized weightlifting machines or free weights. The duration of sessions ranged between 20 and 75 minutes, twice to five times a week (12, 25, 29, 31, 35).

### 3.5 Impact of physical exercise intervention on glycemic control and other variables

HbA1c levels significantly decreased in the included reports, except for two of them (31, 39). Similar improvements in HbA1c levels were found after HIIT and combined training when compared to aerobic and resistance training (28). There are divergent results regarding the similar improvement induced in glycemic control after HIIT and moderate-intensity exercise (34) or combined exercise (25, 29) in contrast to the improvements achieved only after HIIT but not moderate-intensity aerobic training (32, 40, 41). Only one study compared high-intensity and moderate-intensity exercise and found that HbA1c levels were reduced in both groups, with a greater reduction after high-intensity training (33) (**Table 4)**.

**Table 4:** Impact of physical exercise intervention protocols on glycemic control, lipid profile, blood pressure, anthropometric indicators, body composition, physical fitness, and medications in use.

Author, year	HbA1c	Fasting blood glucose	Lipid profile	Blood Pressure	Changes in antidiabetic medication regiments associated with training	Anthropometric measures/body composition	Physical Fitness
Alvarez et al., 2016 (27)	Reduction on HbA1c (~12%, 0.9 ± 0,1%).	Reduction in fasting glucose (~14%, 19.8 ± 1.9 mg/dl).	Increase in HDL (10.1 ± 1.1mg/dL) and decrease in triglycerides (22.9 ± 3.4mg/dL)	Systolic blood pressure reduced after training (-3.7 ± 0.5 mmHg)	After training, seven participants reduced their daily dosage of metformin and glibenclamide, and three participants no longer needed antihypertensive drugs.	Reductions in body weight (-1.6 ± 0.2 kg), BMI (-2.1 ± 0.3%), waist circumference (-4.1 ± 0.6 cm), and skinfold thickness (-18.6 ± 1.4 mm).	Improvement in cardiorespiratory performance was assessed by the reduction of 9.8% (± 1.0%), in the time to walk 2km.
Andrade et al., 2016 (37)	Reduction on HbA1c (~16%, 0.9%)	Fasting glucose did not reduce significantly after intervention.	Not assessed	Not assessed	Not assessed	Not assessed	Not assessed
Bacchi et al., 2012 (28)	Similar reductions in both groups on HbA_1c._ Aerobic Group: -0.40% (CI 95%: -0,61 to -0,18); Resistance Group: -0.35% (CI 95%: -0,59 to -0,10).	Aerobic Group: -15.2 mg/dL (IC95%: -29.8 to -0.57) Resistance Group: -12 mg/dL (IC95%: -23.4 to -0.5).	Aerobic and Resistance groups had similar mean changes on HDL (Aerobic: 2,9, -0.28 to 6.1; Resistance: 1.3, -1.1 to 3.8) and triglycerides (Aerobic: -27.8, -57.5 to 1.7; Resistance: -23.9, -49.5 to 1.6)	Aerobic and Resistance groups had similar mean changes in: Systolic blood pressure (Aerobic: -6.8, -15.5 to 1.8; Resistance: -5.1, -12.4 to 2.3) and Diastolic blood pressure (Aerobic: -4.6, -9.3 to 0.06; Resistance: -2.0, -6.6 to 2.6)	No significant changes were registered. Antidiabetic medication was reduced in four subjects in the aerobic training group and in two subjects in the resistance training group.	Aerobic and Resistance groups had similar reductions in body weight, waist circumference, total body fat, truncal fat, and visceral and subcutaneous adipose tissue. Also, similar increases in lean mass were shown.	VO_2_ Peak: Aerobic group (+4.0 (CI95% 2.7 to 5.3) had a mean chance increased twice as high as the Resistance group (+2.1 (CI95% 0.6 to 3.5). Both groups increased from baseline. Strength: only the resistance group had increased strength
Banitalebi et al., 2019 (29)	Reduced after HIIT (~18.9%) and Combined training (~13.1%)	Fasting blood glucose reduced after HIIT training (~34.6%) and Combined training (~23.7%).	Not assessed	Not assessed	Not informed	No significant reduction in weight, BMI, or percentual of body fat from baseline to post-training.	Not assessed
Banitalebi et al., 2021 (25)	Reduction after HIIT (d = -1.82, IC95%: -2.04 to -1.59) and Combined training (d = -1,18, IC95%: -1,38 to -0,97).	Fasting blood glucose reduced after HIIT training (d= -1.67 (CI: -1.89 to -1.45).	Triglycerides reduction only for the HIIT group (-54,14 mg/dL, d=-0.93, IC95%: -1.13 a -0.73).	Not assessed	No significant changes were registered.	Reduction on waist circumference for both groups: HIIT (-10.14 cm; d = -1.15, IC95%: -1.35 to -0.94) e Combined (-6.14 cm; d = -0.78, IC95%: -0.98 to -0.59).	Not assessed
Cassidy et al., 2019 (24).	Reduction of 2.8 mmol/mol (-0.26%) after HIIT training.	Not assessed	Not assessed	No significant changes were registered.	Not informed	No significant reduction was found in weight, BMI, or percentual of body fat from baseline to post-training.	Not assessed
Chien, et al., 2022 (36)	HbA1c levels were decreased significantly.	Not assessed	No significant changes were registered for HDL cholesterol, LDL cholesterol, or triglycerides after the intervention.	Not assessed	No significant changes were registered.	Calf circumference and appendicular skeletal muscle mass increased after resistance training.	Grip strength increased after resistance training. No significant improvement was found in lower limbs muscle strength
Gholami et al., 2020 (30)	HbA1c levels were decreased after training (-1,1%).	Reduction of 17% (-34 mg/dl) on fasting glucose after training.	Not assessed	Not assessed	Not assessed	Not assessed	Not assessed
Kong et al., 2022 (38)	HbA1c levels were decreased after training (-27,8%).	After aerobic training fasting glucose (-17%) and postprandial two hours glucose (-37.5%) reduced	Not assessed	Not assessed	Not assessed	Body weight, BMI, waist circumference, hip circumference, and visceral fat area were reduced after aerobic training.	Not assessed
Li et al., 2012 (39)	HbA1c levels did not reduce significantly after interventions.	Fasting glucose did not reduce significantly after interventions.	HDL LDL, and triglyceride levels did not reduce significantly after interventions.	Systolic blood pressure decreased after both low-intensity and high-intensity exercise training	Not assessed	BMI reduced after low-intensity (~2,3%) and high-intensity training (~1,9%). Body fat is reduced after low-intensity (~3.6%) and high-intensity training (~3,3%).	VO_2_peak increased for both groups
Magalhães et al., 2019 (31)	HbA1c levels did not reduce significantly after interventions.	Fasting glucose did not reduce significantly after interventions.	Not assessed	Not assessed	Not assessed	Gynoid fat index and whole-body lean tissue improve post-training for both groups. BMI, waist circumference, whole body fat, and abdominal fat did not reduce for both groups.	VO_2_peak increased only for the moderate-intensity continuous + resistance training group.
Mitranun et al., 2014 (32)	HbA1c levels reduced significantly only after HIIT training (~10%; -6 mmol/mol).	Fasting glucose decreases after moderate-intensity (12,9%; -0,99mmol/L) and HIIT (~13,7%; -1,05mmol/L) training.	Total cholesterol reduced after HIIT training (~10%; -0.49 mmol/L). HDL (HIIT: ~29.2%, 0.31 mmol/L; Moderate: ~5.84%, 0.08 mmol/L) and LDL (Moderate: ~16.7%; -0.57 mmol/L; HIIT: ~21.9%; -0.73 mmol/L) cholesterol improved after both trainings. Triglyceride did not change after training.	Systolic blood pressure decreased after HIIT training (~9%, -12 mmHg).	No changes occurred in the dosage of medications.	Body mass (~3.2%, -2.1kg) and BMI (~3.7% (-1.1 kg/m^2^) reduced after HIIT training. Both training reduced Body fat (%) reduced (HIIT: ~6.7%, -2.2%; Moderate-intensity: ~.7%, -2.6%) and waist-to-hip ratio (HIIT: ~2.1%; -0.02 cm; Moderate-intensity: ~2.1%; -0.02 cm)	VO_2_max increased in after HIIT (~25.2; 6.1mL/kg/min) and moderate-intensity training (~13.9%; 3.3mL/kg/min). Leg extension strength increased in both exercise groups. Knee flexion strength increased only after HIIT training.
Nicolucci et al., 2012 (12)	The exercise produced significant improvements in HbA1c (−0.30%; −20.2 nmol/mol, 95%CI: −18.1, −22.4).	Not reported	HDL (0,096 mmol/l, IC95%: 0.057, 0.137) and LDL (−0.249 mmol/l, IC95%: −0.412, −0.085) improved after intervention.	Systolic blood pressure (−4,2 mmHg, IC95%: −6.9, −1.6) and Diastolic blood pressure (−1,7 mmHg, IC95%: −3.3, −1.1) reduced after intervention.	Not assessed	BMI (−0.78 kg/m^2^, IC95%: −1.07, −0.49) and waist-circumference (-3,6 cm, IC95%: -4.4, -2.9) reduced after intervention.	Not reported
Pandey et al., 2017 (33)	HbA1C levels reduced in both groups, despite greater reduction after BURST training (BURST: -10% ± 4% Moderate-intensity: -3% ± 3%)	Not assessed	The lipidic profile had a greater improvement after BURST training. Triglycerides (BURST: -25% ± 14%, -0,17 ± 0,28 mmol/L; Moderate-intensity: -5% ± 9%, -0.86 ± 0.54 mmol/L). HDL: BURST: 23% ± 14%, 0.14 ± 0.08 mmol/L; Moderate-intensity: 3% ± 5%, 0.02 ± 0.03mmol/L). LDL: BURST: -0.37 ± 0.18, Moderate-intensity: -0.16 ± 0.13)	Not assessed	Not assessed	BMI decreased more after BURST training (-2.1 ± 1.2kg/m^2^) than in after moderate-intensity training group (-0.7 ± 0.7 kg/m^2^).	Fitness improved after both BURST (1.27 ± 0.63) and Moderate-intensity (0.24 ± 1.39) training, despite more significative improvements after BURST.
Sabag et al., 2020 (34)	HbA1c decreased after Moderate-intensity (-0.3% ± -0.3%) and HIIT (-0.3% ± -0.3%) training. There was no difference between interventions.	Fasting glucose reduced after training (Moderate-intensity: ~1.3%, -0.1 mmol/L; HIIT: ~4.3%, -0.3 mmol/L)	No significative changes were registered for Total cholesterol, HDL cholesterol, LDL cholesterol, or triglycerides after the intervention.	Not assessed	Not assessed	Reduced waist circumference (Moderate-intensity: -3.0 cm; HIIT: -4.2 cm), with no difference between interventions.	Similar improvement in cardiorespiratory fitness after moderate-intensity (2.3 ± 1.2 mL/kg/min) and HIIT (1.1 ± 0.5 mL/kg/min) interventions.
Stoa et al., 2017 (40)	HbA1c reduced (-0.58%) after HIIT training, while no change was found after moderate-intensity training. There is a significant difference in change between training groups.	Not assessed	Triglycerides (-0.21 ± 0.40 mmol L^-1^) and HDL (0.09 ± 0.16 mmol L-1) were reduced only after moderate-intensity training. No changes on Cholesterol and LDL for both groups.	Systolic blood pressure was reduced after moderate-intensity training (-12 ± 21 mmHg). Diastolic blood pressure was reduced both after moderate-intensity (-8 ± 12 mmHg) and HIIT training (-6 ± 8 mmHg).	Four participants (two in each group) reduced the insulin or medication	After HIIT reductions were found in: Body weight (-17 ± 1.8 kg), BMI (-0.6 ± 0.6 kg m2), Body Fat (-2.7 ± 2.3%), waist circumference (-2 ± 3cm), and hip circumference (-1 ± 2cm). After moderate-intensity only reductions in Body Fat (-1.8± 0.9%), waist circumference (-2 ± 1.6cm), and hip circumference (-1 ± 1.8 cm) were detected. Body weight and BMI were significantly different between intervention groups with the greatest improvement after HIIT intervention.	VO2 max increased by 19% (0.45 ± 0.22 L min-1); after HIIT training.
Winding et al., 2018 (41)	HbA1c levels reduced only after HIIT training (~1.5%, 0.1%).	Fasting blood glucose reduced only after the HIIT training (~8%, 0.7 mmol/L)	No significant changes were registered.	No significant changes were registered.	No changes occurred in the dosage of medications.	After both interventions body mass and android fat mass were found to be different from the control group. Comparison pre vs post interventions showed improvements after HIIT on whole body mass (~1,2%, - 1kg), android fat (~3,2%, - 0,2kg), and visceral fat mass (~11,8%, -0,2kg). After Moderate-intensity intervention improvements on gynoid fat mass (~5%, -0,2kg) was found	Both trainings increased VO_2_peak, despite a greater increase after HIIT training (20% ± 20%) compared to moderate intensity training (8% ± 9%).
Yang et al., 2017 (35)	HbA1c reduced after training (-0.7%), with no differences between interventions. However, the RT1 group showed the most beneficial reduction in HbA1c (-1.1%) compared to RT2 (-1%) and RT3 (-0.4%).	Fasting glucose did not improve after training. However, it was different between RT1 and RT3 training groups (RT1-RT3: -1.07; 95% CI: -2.13 to -0.2).	No significant changes were registered.	Not assessed	Not assessed	Post-training reductions were identified in BMI (~1.7%, -0.53kg/m^2^), fat mass (~4.1%, -1.13kg), body fat (~3.1%, -1.01%), and body mass (~1.2%, -0.98kg), with no differences between groups.	VO2 max increased after training (~16.9%, 3.64 ml kg-1 min-1), with no differences between groups.

HIIT, treino intervalado de alta intensidade; BURST, exercício contínuo de alta intensidade; TR, treino resistido. Only statistically significant changes are presented.

A significant reduction in fasting blood glucose was reported after HIIT, moderate-intensity aerobic, resistance, or combined training (25, 27–30, 32, 34, 38, 41). Similar reductions in fasting blood glucose were reported when comparing aerobic and resistance training (28). Comparisons between HIIT and moderate-intensity or combined interventions show divergent results, with some studies showing similar improvements between intervention groups (29, 32), while others provide evidence to support that HIIT leads to superior results in reducing fasting glucose (25, 34, 41). Four studies reported non-significant results (31, 35, 37, 39).

HIIT induced improvements in blood pressure, anthropometric measures, body composition, and physical fitness (25, 27, 32, 34), but not under an unsupervised training program (24). HIIT had different impacts on the lipid profile, resulting in improvements (27, 32), but also no significant changes were reported (21, 26–28). Two studies found no significant changes in blood pressure (24, 41).

Only seven of the studies (38.9%) included in the present review assessed the impact of physical exercise interventions on the dosage of antidiabetic medication, reporting reductions in medication (27, 40), but also no exercise-induced modification in medication prescription (25, 28, 32, 36, 41).

Positive modifications in anthropometric measures, body composition, and physical fitness were described with similar results after the different intervention programs, most of them without clinically significant differences. HIIT and continuous high-intensity aerobic interventions showed superior improvements in aerobic fitness than moderate-intensity protocols. Coherently, aerobic training and muscular strength training were associated with improvements in cardiorespiratory fitness and muscular fitness, respectively.

## 4 Discussion

This study aimed to produce a literature synthesis, through a rapid review, on the effects of aerobic, resistance, and combined physical training variables on glycemic control in adults with type 2 Diabetes Mellitus. There is a strong body of evidence on the therapeutic effect of physical exercise on the prevention and treatment of T2DM (13, 16, 21, 42). However, previous studies have reported important heterogeneity in adherence rates and a low rate of patients with diabetes meeting the minimum amount of exercise recommended by guidelines (43, 44). The adaptation of physical exercise variables to the patient’s clinical needs, disease-specific symptoms, personal preferences, and time availability is important for long-term adherence to the exercise program (21, 43) and to an active lifestyle.

The studies included in the present review reported a significant reduction in HbA1c, regardless of the modality, duration, and frequency of exercise. However, regarding intensity one study investigated a low-intensity aerobic exercise and did not reach significant reductions in HbA1c, fasting glucose, or lipid profile (39). Other evidences found improved insulin sensitivity after an acute bout of low-intensity exercise, lasting for less than 24 hours (45) and also after a single low-intensity resistance training session (46). In this context, it is necessary to consider the importance of an active lifestyle and of even small amounts of exercise (8), and that low-intensity exercise may be an alternative for those patients at high risk for acute cardiovascular events, undergoing cardiac rehabilitation (21).

Superior effectiveness of HIIT for glycemic control was reported in the included studies in comparison to moderate-intensity (32, 40, 41) or combined training (25, 29, 32), except by one study that reported comparable effectiveness between HIIT and moderate-intensity training (34). Additionally, included studies that compared the metabolic benefits of HIIT to non-exercise control groups found improvements in HbA1c after interventions (24, 27, 32). These results add new and positive insight into previous inconclusive meta-analysis results in comparing HIIT *vs* moderate intensity exercise to reduce HbA1c levels (47), suggesting this modality as an efficient option for T2DM patients.

Only two reports from the same study (25, 29) compared a combined exercise program with another modality (HITT) and found the greatest percentual reduction after HIIT, despite a large effect size after both combined training or HITT on HbA1c reduction. This is in contradiction with previous studies showing that the greatest reduction in HbA1c is observed when using combined exercise compared to the other modalities alone (13, 48, 49). However, considering the limited information here available, it remains inconclusive if the combined training is more effective for glycemic control.

Resistance training comparably reduced HbA1c levels compared to aerobic exercise, with no statistical difference between the two groups (28), which supports previous findings (6, 29). However, a previous study (50) showed that while resistance exercise was effective and resulted in a -0.34% reduction in HbA1c with a large effect size, these reductions were not observed in individuals with a mean HbA1c level ≤ 7.5% (non-significant effect size), suggesting that a resistance exercise program alone may not effectively contribute to strict glycemic control in individuals with HBA1c values closer to the ideal range.

Previous results support that improvements in glycemic control induced by exercise are greater in individuals with higher HbA1c levels at baseline (51). We found mixed results on that, once the included studies that fail to find a reduction in HbA1c included participants within or near the recommended target of <7.0% for glycated hemoglobin baseline (31, 39, 40), but others got improvements after moderate-intensity, resistance, HIIT, and also combined training (12, 24, 28, 34, 37). In only one study with near to target patients HIIT, but not moderate-intensity exercise, promoted improvements in HbA1c (41). This result suggests that there are no preferential modalities to be indicated for patients with adequate glycemic control.

Not all the studies included in this review (30–32, 34, 36) meet the minimum of 150 minutes of moderate-intensity exercise per week or 75 minutes of combined moderate and high-intensity exercises, but all of them, except one (12) performed the minimum frequency recommended. The weekly frequency is an important factor to improve glycemic control and decrease cardiovascular risk in patients with T2DM (52). The recommendation that exercise should be performed with no more than 2 consecutive days between bouts of activity (13) is due to the transient exercise-induced sensitivity to insulin in individuals with T2D for up to 48 hours (21).

Structured exercise regimens exceeding 150 minutes per week have shown greater reductions in HbA1c levels compared to those with 150 minutes or less per week in individuals with type 2 diabetes (53). Two included papers from the same study (25, 29) were a shorter intervention (10 weeks) and found no positive impacts of the exercise on fasting glucose, lipidic profile, on medication dosage, and some measures of body composition. Moreover, the effects of physical exercise on HbA1c and BMI are found to be associated with interventions lengths, with an incremental decrease in HbA1c of 0.009 to 0.043% for each additional week of physical training, underscoring the importance of adherence to exercise for a sustained lifestyle change in achieving health improvements (41). However, a recent meta-analysis suggests that interventions longer than 12 weeks do not induce additional benefits on HbA1c (54). Taken together the results of the included studies reinforce the need for regularity and spacing between physical training sessions, but also the need for progression of the intervention intensity for adequate management and glycemic control (55).

Individuals with T2DM show a reduced maximal aerobic capacity which increases with disease duration. Although associations between poor glycemic control e reduced cardiorespiratory fitness had been established, the mechanisms are not fully understood (56). On the other hand, it is known that physical exercise may improve physical fitness in adults with diabetes (56). Cardiorespiratory fitness improvements were found in all studies included in the present review. As expected, the magnitude of the improvements was related to training modalities, with greater improvements due to aerobic and HIIT training, but also found after resistance and combined training, and in response to either interval, low-intensity, moderate- and high-intensity training (27, 28, 32–35, 39, 40). Divergent results were found showing improvements only after moderate-intensity or HIIT, but not both (31, 40). Greater improvement after HIIT or high-intensity continuous aerobic exercise *vs* Moderate-intensity was found (32, 33, 41). Strength was improved after resistance training (28, 32, 36) and also after HIIT and Moderate-intensity exercise in the lower limbs (32). Facing the diabetes-associated decline in fitness and the evidence of the improvement associated with aerobic capacity training and strength, it is mandatory its inclusion in exercise programs dedicated to individuals with diabetes.

Aerobic training interventions lead to improvements in cardiorespiratory fitness, with evidence of greater effectiveness of high-intensity interventions. Among the reviewed studies, only 50% reported objective monitoring of the exercise session by using heart rate monitors. By not monitoring heart rate, the accuracy of exercise intensity is reduced, and an important bias is added to the protocol analysis. High-intensity aerobic exercise has demonstrated superiority over moderate-intensity exercise in improving physical fitness measures such as VO_2_max and anthropometric indicators like body weight and BMI. The clinical implications of high-intensity aerobic exercise’s effectiveness in enhancing physical fitness are noteworthy, as VO_2_max serves as a predictor of cardiovascular risk, and its improvement is associated with decreased morbidity and mortality from cardiovascular diseases as well as a lower prevalence of diabetes (57)

We found that moderate-intensity and high-intensity exercises promoted reductions in HbA1C, but high-intensity seems to be more effective in regulating fasting glucose. In fact, only two studies did not report positive results on that variable. One of them (39) compared high *vs* low-intensity aerobic training in participants with baseline levels of HbA1C lower than 7.0% and overweight. The second one (31) compared HIIT + resistance training *vs* moderate-intensity + resistance training in obese participants with high MVPA at baseline, but did not control for modifications on medications and compliance with the program.

Improvements in fasting blood glucose levels, lipid profile, blood pressure, and changes in antidiabetic medication regiment are not consistently impacted by exercise modalities on the included studies. HIIT and moderate-intensity interventions most consistently showed positive impacts on fasting glucose levels and lipid profile. Some studies reported divergent results after combined training and no improvements after low-intensity aerobic training and resistance training. It is important to note that only one study was dedicated to investigating low-intensity and resistance training. Mixed results were found regarding modifications in the antidiabetic medication regiment associated with the physical training after HIIT, resistance training, and moderate intensity. Blood pressure reduced after HIIT, low intensity, moderate intensity, high intensity, resistance training, and combined training, but no significant changes after HIIT, moderate intensity were reported. These outcomes measures were not investigated in all included studies, as well, there are important differences in the intervention protocols. This heterogeneity limits the conclusion regarding those parameters. A core outcome set to be used in randomized controlled trials in type 2 diabetes was to provide greater uniformity and comparability between studies and, thus, generate information for clinical practice (58).

Participant adherence to the exercise intervention ranged from 78% to 93%, with no differences regarding modalities. The supervision of an exercise professional can improve exercise adherence and safety, particularly for physically inactive adults and individuals with chronic diseases initiating an exercise program, but individuals with T2DM show adequate adherence under different supervision regiment (43, 59, 60). An essential factor influencing participant adherence is the personal affinity with the chosen modality and the suitability of training to the personal routine, emphasizing the importance of respect for individual preferences in line with the principles of Evidence-Based Practice (50, 61).

Physical exercise is safe for individuals with T2DM. No adverse events or just minor events – including back pain, tendinitis, hypoglycemia, or muscle injuries – were described, and do not raise restrictions or contraindications for the practice of physical exercise by adults with type 2 diabetes. It is recognized that implementing screening protocols beyond routine diabetes care can mitigate the risk of exercise-induced adverse events in asymptomatic individuals with diabetes (15). However, precautions should be taken to avoid that the screening requirements do not lead to unnecessary barriers to initiating an exercise program (62), especially in individuals with limited or not covered by health insurance.

Randomized clinical trials are the gold standard in investigating dose-response, causal relationships, and efficacy of physical training interventions (22). The design of the exercise training clinical trials protocols needs to be detailed planned and reported, including frequency, intensity, type, duration, and progression. While the diversity of the intervention protocols included in this review is an important limitation to the evidence-based practice, its support the effectiveness and safety of the physical exercises, in different modalities and intensities, including HIIT, as non-pharmacological interventions for glycemic control in individuals with type 2 diabetes. The call for action is mandatory to implement large-scale education programs on the prevention of diabetes and public health policies aimed to include well-planned exercise programs as an essential part of the primary care for type 2 diabetes.

## 4.1 Study limitations

This review revealed great variety in the prescription of exercise protocols. Furthermore, a lack of sufficient information regarding the design of exercise interventions, particularly concerning the overall training volume, was observed. This limitation may prevent the assessment of adaptability and responsiveness to the performed exercises.

It is important to acknowledge other limitations, including the inclusion of non-randomized clinical trials and clinical trials with a high risk of bias. Additionally, the studies exhibited heterogeneity in exercise prescription variables, inadequate or non-existent descriptions of exercise progression, and a lack of supplementary information such as exercise adherence, timing, and adverse events.

At least, it is important to note that although our planned PICOT strategy included the population over 18 years of age, with no age limits, young adult, and older adult populations (≥ 65 years of age) are not significantly represented in the included studies. The studies included in this review have an average age group of 56.1 years old, within the minimum 43.1 and maximum of 68 years of age. In this scenario, it is recommended to take caution in extrapolating the information to older adults, especially over 70s, and young adults. Therefore, there is a gap in the literature regarding the impacts of physical exercise parameters as a nonpharmacological strategy for the treatment of people in these age groups with T2DM.

## Data availability statement

The original contributions presented in the study are included in the article/supplementary material. Further inquiries can be directed to the corresponding author.

## Author contributions

NB-T participated in the conception of the study, supervision, data analysis, interpretation of results, and writing. AR participated in the conception of the study, data extraction and analysis, interpretation of results, and writing. JC participated in the data extraction and analysis, interpretation of results, and writing. All authors contributed to the article and approved the submitted version.
